# Role of Metabolic Stress and Exercise in Regulating Fibro/Adipogenic Progenitors

**DOI:** 10.3389/fcell.2020.00009

**Published:** 2020-01-28

**Authors:** Nicolas Collao, Jean Farup, Michael De Lisio

**Affiliations:** ^1^School of Human Kinetics, University of Ottawa, Ottawa, ON, Canada; ^2^Department of Biomedicine, Aarhus University, Aarhus, Denmark; ^3^Department of Cellular and Molecular Medicine, Centre for Neuromuscular Disease, University of Ottawa, Ottawa, ON, Canada

**Keywords:** obesity, metabolic syndrome, FAPs, differentiation, skeletal muscle, physical activity, exercise, mesenchymal stem cell

## Abstract

Obesity is a major public health concern and is associated with decreased muscle quality (i.e., strength, metabolism). Muscle from obese adults is characterized by increases in fatty, fibrotic tissue that decreases the force producing capacity of muscle and impairs glucose disposal. Fibro/adipogenic progenitors (FAPs) are muscle resident, multipotent stromal cells that are responsible for muscle fibro/fatty tissue accumulation. Additionally, they are indirectly involved in muscle adaptation through their promotion of myogenic (muscle-forming) satellite cell proliferation and differentiation. In conditions similar to obesity that are characterized by chronic muscle degeneration, FAP dysfunction has been shown to be responsible for increased fibro/fatty tissue accumulation in skeletal muscle, and impaired satellite cell function. The role of metabolic stress in regulating FAP differentiation and paracrine function in skeletal muscle is just beginning to be unraveled. Thus, the present review aims to summarize the recent literature on the role of metabolic stress in regulating FAP differentiation and paracrine function in skeletal muscle, and the mechanisms responsible for these effects. Furthermore, we will review the role of physical activity in reversing or ameliorating the detrimental effects of obesity on FAP function.

## Introduction

Over the last decades, lifestyle changes in western societies such as diet and physical inactivity are a major global public health problem leading to metabolic syndrome (MetS) (Saklayen, 2018). MetS is a cluster of different conditions including central adiposity, hypertension, insulin resistance, inflammation, and dyslipidemias, among others, which are themselves risk factors for type 2 diabetes (T2D), cardiovascular disease and even increasing the risk of cancer (Manuel et al., 2014). According to the World Health Organization (WHO), obesity (body mass index (BMI) ≥ 30 kg/m^2^) has almost tripled since 1975, such that as of 2016 over 603 million adults and 107 million children are obese (WHO, 2019). This increased incidence of MetS is associated with an increase in the prevalence of musculoskeletal diseases and disorders (Wearing et al., 2006; Collins et al., 2018). Emerging evidence has shown that metabolic complications are positively correlated to reduction of muscle mass, impaired muscle repair, and increase in fibro/fatty tissue accumulation (Akhmedov and Berdeaux, 2013). These pathological changes ultimately result in increased morbidity and disability (Hoy et al., 2014; March et al., 2014).

Underlying these pathological changes are alterations in the heterogeneous stem/progenitor cell populations that reside within skeletal muscle. The regenerative potential of skeletal muscle relies primarily on myogenic stem cells, called satellite cells (MuSCs), residing under the myofiber basal lamina (Wang and Rudnicki, 2011; Bentzinger et al., 2013). Upon muscle injury MuSCs enter the cell cycle, proliferate, and differentiate to repair damaged myofibers, while self-renewing to repopulate the reserve pool (Feige et al., 2018). Recently, a novel mesenchymal cell population of non-myogenic cells, named fibro/adipogenic progenitors (FAPs), has been identified in the skeletal muscle interstitium (Joe et al., 2010; Uezumi et al., 2010). FAPs are critical during muscle regeneration in order to sustain MuSC differentiation via paracrine mechanisms, and to maintain the MuSCs pool (Wosczyna et al., 2019). However, in pathological conditions FAP expansion continues unchecked, resulting in the production of fibro/fatty infiltrations, and impaired myogenesis (Rodeheffer, 2010; Uezumi et al., 2010, 2011; Mozzetta et al., 2013; Dong et al., 2017; Madaro et al., 2018; Stumm et al., 2018). Metabolism plays a crucial role in controlling the fate of progenitor cells, including MuSCs in tissue development, homeostasis, regeneration, and disease (Ryall et al., 2015a; Knobloch et al., 2017; Pala et al., 2018); however, the effects of metabolic stress and the metabolic regulation of FAPs has only recently begun to be explored. As such, the purpose of the present review is to provide an overview of the current state of the literature regarding to the effects of metabolic stress, induced by disease or exercise, on FAP differentiation and paracrine function.

## Connection Between Metabolic Stress and Ectopic Adipose Tissue Accumulation in Muscle

Skeletal muscle is an important tissue for the regulation of whole-body metabolic homeostasis. In most individuals, skeletal muscle comprises 40–60% of the total body mass, accounts for ∼30% of the resting metabolic rate in adult humans (Zurlo et al., 1990), is a key contributor to whole body lipid utilization (Egan and Zierath, 2013), and ∼80% insulin-stimulated glucose disposal (Brüning et al., 1998). Skeletal muscle has a high capacity for substrate oxidation and a relatively high potential for substrate storage (DeFronzo et al., 1981). Under conditions of metabolic stress, detrimental changes to skeletal muscle occur, including muscle loss, intra- and inter-myofibrillar lipid accumulation, and connective tissue deposition. Eventually, these changes lead to a detrimental effect on contractile function and/or metabolic properties of skeletal muscle having an important impact on human health and contribute to insulin resistance (Sakuma and Yamaguchi, 2013). In the obese state, muscle lipid accumulation may occur as a result of insufficient adipose tissue expansion, in which the excess lipid are stored in non-adipose tissue compartments such as liver and skeletal muscle (Szendroedi et al., 2014; Cuthbertson et al., 2017; Czech, 2017; Conte et al., 2019). One result of the ectopic adipose tissue accumulation is the expansion of intramyocellular lipids (IMCLs) located within muscle cells (Sinha et al., 2002; Boesch et al., 2006). Paradoxically, endurance trained individuals also demonstrate an accumulation of IMCLs that are distinguished from the obese state by their location (Samjoo et al., 2013). Whereas in athletes IMCLs provide a local store of substrate for aerobic ATP generation, in persons with obesity, IMCLs are linked to insulin resistance and increased risk of T2D (Kautzky-Willer et al., 2003; Brumbaugh et al., 2012).

Intermuscular adipose tissue is distinguished from IMCLs as the former represents adipocytes that form between muscle fibers and muscle groups (Hamrick et al., 2016). Intermuscular adipose tissue increase with age in humans (Kirkland et al., 2002; Addison et al., 2014) and is highly correlated with a decrease in muscle mass, muscle strength, and insulin-sensitivity (Visser et al., 2005; Miljkovic-Gacic et al., 2008; Delmonico et al., 2009). Similarly, in participants with obesity, intermuscular adipose tissue accumulates and is associated with systemic insulin resistance (Goodpaster et al., 2000; Goss and Gower, 2012). Although not yet directly tested, the local accumulation of intermuscular adipose tissue may impair muscle metabolism by producing high intramuscular concentrations of adipokines, adipose-derived hormones, and free fatty acids. In support of this notion, some studies have reported positive correlations between intermuscular adipose tissue accumulation and the decrease in insulin sensitivity observed during aging and obesity (Goodpaster et al., 1997, 2000; Ryan and Nicklas, 1999; Sachs et al., 2019). Interestingly, the majority of studies on intermuscular adipose tissue accumulation under conditions of metabolic stress have been conducted in humans. Three recent studies indicated that intermuscular adipose tissue accumulates in rodents in both obesity (Khan et al., 2015; Zhu et al., 2019) and aging (Cui et al., 2019). This is relevant because most of the work investigating the mechanisms regulating intermuscular tissue adipose tissue accumulation have been conducted in rodents. Thus, similar responses in human and rodent skeletal muscle have been observed in the limited studies that have evaluated intermuscular adipose tissue accumulation in metabolic stress.

## Fibro/Adipogenic Progenitors as the Cellular Source of Intermuscular Adipose Tissue

Fibro/adipogenic progenitors are muscle-resident non-myogenic progenitors of mesenchymal origin, which express stem cell antigen 1 (Sca-1), platelet-derived growth factor receptor α (PDGFRα), and also high levels of CD34 (Joe et al., 2010; Uezumi et al., 2010, 2014). FAPs are distinct from MuSCs as they lack Pax7 expression (Joe et al., 2010; Uezumi et al., 2010, 2014). FAPs have been defined as multi-potent progenitors, residing on the abluminal side of the capillaries in the interstitial spaces between the myofibers in both humans and mouse skeletal muscle (Joe et al., 2010; Uezumi et al., 2010; Arrighi et al., 2015). These cells are defined by their ability to differentiate into fibroblasts, adipocytes, and osteoblasts, and originate from a non-myogenic (Myf5-) cell population which is supported by their lack of myotube formation *in vitro* (Joe et al., 2010; Uezumi et al., 2010). Following muscle injury, FAPs transiently become activated, proliferated, and expand (Lemos et al., 2015; Wosczyna et al., 2019). Via primarily paracrine mechanisms, FAPs promote MuSC proliferation (Fiore et al., 2016) and differentiation (Joe et al., 2010; De Lisio et al., 2014; Zou et al., 2015; Contreras et al., 2016; Dammone et al., 2018; Madaro et al., 2018), thus participating in muscle repair. Conversely, in pathological conditions characterized by myofiber damage or atrophy, FAPs undergo unchecked expansion and differentiation causing fibrosis, fat deposition an impaired myogenesis (Lemos et al., 2015; Dammone et al., 2018; Madaro et al., 2018).

Metabolic stress has been linked to FAP accumulation and fibro/adipogenic differentiation (Dammone et al., 2018; Gorski et al., 2018; Kang et al., 2018; Buras et al., 2019). Using several different genetic and diet-induced mouse models of diabetes, Mogi et al. (2016) demonstrated that ectopic adipocyte accumulation in skeletal muscle was derived from PDGFRα^+^ progenitors. Similarly, Arrighi et al. (2015) isolated a population of FAPs, identified as CD56^–^CD15^+^/PDGFRα^+^, that formed functional adipocytes *in vitro*. These FAP-derived adipocytes may have reduced insulin sensitivity compared to conventional adipocytes, as indicated by lack of phosphorylation of insulin receptor, suggesting that accumulation of FAP-derived adipocytes may contribute to a compromised peripheral insulin sensitivity (Arrighi et al., 2015). However, given the relatively small contribution of intermuscular adipose tissue relative to whole body adipose depots, the negative effects of intermuscular adipose tissue on glucose disposal is likely via secondary mechanisms that reduce the ability of myofibers to uptake glucose. Similar to findings in limb skeletal muscle, 6 months of high-fat feeding induced FAP proliferation, increased adipocytes, and type I collagen-depositing fibroblasts in the diaphragm leading to respiratory dysfunction (Buras et al., 2019). Together, these data indicate that in obesity and related metabolic disorders, FAPs directly contribute to intermuscular adipose tissue accumulation in skeletal muscle.

Several potential mediators of the effects of obesity on FAP differentiation have been investigated. Adipokines released from expanded adipose tissue such as thrombospondin 1 (THBS1), was increased in obese mice and promoted FAP proliferation (Buras et al., 2019). Similarly, TGFβ which is produced in many organs including adipose tissue (Lee, 2018) controls FAP proliferation and differentiation to a fibrogenic lineage *in vitro* (Ito et al., 2013; Lemos et al., 2015). Conversely, inhibition of PDGFRα and TGFβ signaling resulted in reduced FAP number and a reduction in collagen deposition (Ieronimakis et al., 2013; Ito et al., 2013; Lemos et al., 2015; Fiore et al., 2016). Thus, several adipocyte-derive factors increase FAP adipogenesis, indicating a direct mechanism whereby adipose tissue expansion in obesity may stimulate intermuscular adipose tissue accumulation.

In contrast to adipokines, factors synthesized by myofibers play an important role in limiting adipogenesis during muscle regeneration. Nitric oxide (NO), which is increased in response to muscle injury and exercise, inhibits FAP adipogenic differentiation by down-regulation of the peroxisome proliferator-activated receptors gamma (PPARg) (Cordani et al., 2014). Marinkovic et al. (2019) showed that suppression of myofiber-derived NOTCH signaling via inhibition of γ-secretase or by interfering with the expression of NOTCH stimulates FAP differentiation in a dose-dependent manner, whereas activation by the NOTCH ligand DLL1 leads to significant inhibition of adipogenesis in *mdx* mice. Kopinke et al. (2017) demonstrated a critical role of cilia in modulating the adipogenic fate of FAPs by controlling the activity of the Hedgehog signaling pathway. Pharmacological inhibition of matrix metalloprotease (MMP)-14 represses C/EBPδ and PPARγ in FAPs by way of cilia Hedgehog signaling and this reduces the adipogenic fate of FAPs. As a result, this enhanced muscle regeneration during acute muscular injury and in a model of muscular dystrophy (Kopinke et al., 2017). Collectively, these data indicate that regenerating muscle releases several factors that inhibit FAP adipogenesis, providing a potential mechanism whereby exercise-induced muscle damage may prevent ectopic intermuscular adipose tissue accumulation under metabolic stress.

Cell metabolism is also a driver of mesenchymal progenitor cell fate during differentiation. For instance, during induction of adipogenesis mesenchymal progenitors need to enhance reliance on oxidative phosphorylation in order to continue differentiation into pre- and mature adipocytes (Shyh-Chang et al., 2013). This may explain why incubating fibroblasts from human skeletal muscle with fatty acids is a potent inducer of adipogenesis (Agley et al., 2013). Similarly, generation of osteoblasts is also associated with high reliance on oxidative phosphorylation. In contrast, fibrogenesis and chondrogenesis seems to require utilization of glycolysis during differentiation (Shyh-Chang et al., 2013; Zhao et al., 2019). FAPs from regenerating *mdx* muscle have an increase in glycolytic proteins and a reduction of mitochondrial proteins compared to control mice (Marinkovic et al., 2019) resulting in *mdx* FAPs favoring glycolysis over oxidative metabolism (Reggio et al., 2019). Interestingly, these metabolic changes were associated with greater proliferative capacity and adipogenic potential *in vitro* which was reversed by inhibiting glycolysis and forcing oxidative metabolism (Reggio et al., 2019). This impaired metabolic phenotype was reversed *in vivo* by providing a short-term high fat diet which stimulated oxidative metabolism in FAPs (Reggio et al., 2019). Conversely, long-term high fat diet, and obesity are associated with increased muscle adiposity and fibrosis (Goodpaster et al., 2000). Hogarth et al. (2019) identify FAPs and their adipogenic differentiation as a major contributor to dysferlin-deficient muscle loss in limb-girdle muscular dystrophy 2B (LGMD2B); a disease associated with mitochondrial dysfunction (Vincent et al., 2016). Together, these interesting findings indicate that mitochondrial function and metabolism are important regulators of FAP fate, and that the FAP response to metabolic stress may be distinct from other interstitial cells in skeletal muscle or from mesenchymal cells in different tissues. Further, they suggest that FAP fate may be regulated by substrate availability, which provides novel areas for therapy.

The effects of exercise on FAP differentiation have yet to be fully elucidated. Endurance exercise training is associated with an increase in IMCLs, but not intermuscular adipose tissue (van Loon et al., 2004; Dubé et al., 2008). Furthermore, acute resistance and endurance exercise is known to increase muscle extracellular matrix synthesis with a similar increase in breakdown resulting in matrix remodeling without excessive accumulation, unlike pathological conditions (Martinez-Huenchullan et al., 2017). Using a preclinical model of radiation exposure, recent work from our group showed a differential FAP response of obese and exercise trained mice (D’Souza et al., 2019). Interestingly muscle fibrosis and adipose tissue accumulation were higher in high fat-fed mice with no effect of treadmill exercise. However, we were not able to distinguish between intermuscular and intramyocellular adipose tissue in our analyses. Conversely, FAP content trended to increase in high fat-fed, sedentary mice, and trended to be reduced in high fat-fed, exercise trained mice (D’Souza et al., 2019). While these results are preliminary, they suggest that exercise training may inhibit FAP expansion in obesity. Similarly, Zeve et al. (2016) reported that endurance exercise suppressed adipogenic progenitor proliferation and differentiation into mature adipocytes *in vitro* and suppressed adipogenesis *in vivo* in mice fed a high fat diet. These effects were mediated in part through secretion of R-spondin 3 from slow myofibers, which may activate Wnt signaling to suppress adipogenesis (Zeve et al., 2016). In the bone marrow, exercise training and mechanical forces inhibit adipogenic differentiation of mesenchymal stromal cells (Emmons et al., 2017; Rubin et al., 2018), a population similar to skeletal muscle FAPs. Future work should investigate if similar effects of exercise are present in skeletal muscle FAPs. Under conditions of metabolic stress, exercise may directly regulate oxidative capacity of FAPs by stimulating mitochondrial biogenesis as it does in skeletal muscle, or counteract the pro-adipogenic effects of adipokines by increasing secretion of anti-adipogenic factors from myofibers.

## The Relationship Between Metabolic Stress and the Fap Secretome

Fibro/adipogenic progenitors exert much of their functional effects on muscle regeneration and repair via responding to and secreting paracrine factors in their local microenvironment (Joe et al., 2010; Lemos et al., 2015). The most complete list of factors that are secreted by FAPs, and that FAPs respond to have been reviewed elsewhere (Biferali et al., 2019). Interestingly, despite the importance of paracrine factor signaling on FAP function, relatively little attention has been paid to the role of metabolic stress on the FAP secretome. One myogenic paracrine factor that has received some attention in the literature is follistatin (Mozzetta et al., 2013; Reggio et al., 2019). Follistatin is a myostatin inhibitor, and thus promotes muscle growth (Lee and McPherron, 2001) and glucose uptake (Han et al., 2019). Recent studies determined that FAPs are a major source of follistatin in skeletal muscle, and FAP-derived follistatin is a key mechanism responsible for FAP-induced myoblast differentiation (Mozzetta et al., 2013). Further, HDAC inhibitors blocked adipogenic differentiation of FAPs and improved their ability to promote differentiation of MuSCs, through upregulation of the soluble factor follistatin in early stage, but not late stage, *mdx* mice; which are also characterized by metabolic defects (Mozzetta et al., 2013). Using the same *mdx* mouse model of muscular dystrophy, Reggio et al. (2019) demonstrated that follistatin is diminished in FAPs due to reduced β-catenin signaling. However, short-term high fat diet feeding reversed this defect by increasing β-catenin levels which promoted IGF-1 and follistatin expression and release leading to improved ability of FAPs to support myogenesis and muscle regeneration (Reggio et al., 2019). Thus, short-term metabolic reprograming of FAPs, via high fat diet feeding may ameliorate some of the regenerative defects observed in dystrophic mice (Reggio et al., 2019). Similarly, recent work from our laboratory using a model of *in vivo* radiation exposure demonstrated that high fat feeding increased levels of phosphorylated NF-κB, particularly in interstitial nuclei (D’Souza et al., 2019). However, it was not evident if this marker of increased interstitial NF-κB activation was localized specifically to FAPs (D’Souza et al., 2019). In the context of aging, Lukjanenko et al. (2019) determined that diminished FAP secretion of the matricellular protein WNT1 inducible signaling pathway protein 1 (WISP-1) underlies the impairment in MuSC regenerative capacity. However, in this same study, the authours determined that adipogenic differentiation of FAPs is diminished (Lukjanenko et al., 2019). Thus, the signals regulating FAP function may differ between aging and obesity. These data indicate that in models associated with metabolic dysfunction, short-term high fat feeding, can increase paracrine factor secretions from FAPs, which contrast some of the detrimental effects of long-term high fat feeding on FAP differentiation. Mechanistically, it will be important to determine if the specific composition of the diet is relevant to regulating the FAP secretome, or if alterations in FAP paracrine factor secretion are due to increased adiposity associated with the diet, and what regulates the apparent differential responses of FAP differentiation/secretome to long- versus short-term high fat feeding.

Exercise, and exercise-induced signals, have been suggested to alter FAP paracrine function (Boppart et al., 2013). Exercise used to induce a physiologically relevant adaptive response has been shown to increase FAP content in humans (Farup et al., 2015), and a heterogeneous population of muscle mesenchymal stromal cells which contains FAPs in mice (Valero et al., 2012). In these studies, increased FAP content was associated with increases in MuSCs and an enhanced muscle adaptive response to exercise (Valero et al., 2012; Farup et al., 2015). Using a model of *in vitro* “exercise” where isolated muscle mesenchymal cells, including FAPs, were exposed to mechanical forces, it was determined that the strained cells produced a mix of paracrine factors that enhanced myoblast proliferation *in vitro* (De Lisio et al., 2014). Further, mechanical strain applied to these stromal cells *in vitro* prior to injection into muscle *in vivo* improved the muscle response to exercise (Huntsman et al., 2013, 2018; Zou et al., 2015). The relative contribution of FAPs to the overall secretome of the heterogeneous population of muscle-derived mesenchymal cells at rest and after exercise remains to be determined.

## The Effects of Metabolic Stress on Fap Activation

In relation to obesity and T2D one of the most obvious changes in the systemic environment is the chronically elevated levels of metabolic substrates such as glucose and triglycerides. Activation (exit from quiescence) and first cell division of stem/progenitor cells (e.g., MuSCs or FAPs) has in recent years been described as a highly unique process compared to the following (second, third, etc.) cell divisions (Rodgers et al., 2014; Liu et al., 2018). For instance, in mice time-to-first division in MuSCs is approximately 60 h, whereas the following divisions are approximately four times faster. Although not well-described in FAPs yet, we speculate that the same may be true for FAPs since most of these also reside in a quiescent/non-cycling state in normal mouse muscle (Joe et al., 2010; Rodgers et al., 2014). The time before first cell division is characterized by a major increase in cell size, anabolic signaling, mitochondrial content and ATP levels (Rodgers et al., 2014). In MuSCs these changes have been associated with an increased reliance on glycolysis as well as increased autophagic flux likely to support sufficient generation of ATP and biomass to prepare for the cell cycle (Tang and Rando, 2014; Ryall et al., 2015b). In contrast, genetic deletion of major enzymes involved in this anabolic process (e.g., mTORC1) or blocking autophagy delays the cell cycle entry (Rodgers et al., 2014; Tang and Rando, 2014; Ryall et al., 2015b). Glucose and related insulin signaling are major upstream regulators of these pathways making them likely candidates for perturbing the balance between maintaining and exiting quiescence. Although there is yet no direct evidence linking changes in substrate availability in the FAP niche to activation of FAPs it is interesting to note how the major FAP regulator, platelet-derived growth factor (PDGF), is driving changes in cell metabolism. For instance, PDGF treatment of muscle FAPs leads to increased cell proliferation as well as transcriptional changes related to glycolysis (Mueller et al., 2016). Moreover, PDGF signaling in mesenchymal progenitors or fibroblasts from other tissues is known to increase reliance on glycolysis and thereby also increasing lactate production (Ran et al., 2013; Xiao et al., 2017). In fact, fibrosis in multiple tissues is associated with increased glycolytic flux compared to non-fibrotic areas and blocking glycolysis seems to ameliorate some of these pathological events and reduce extracellular matrix accumulation (Zhao et al., 2019). This suggest that increased glycolysis is not merely a result of cell activation, but likely have a causal relationship to cell activation. Collectively, evidence is accumulating that cell metabolism is intimately involved in fibrosis development, in skeletal muscle potentially through priming FAPs for exiting quiescence. Since the normal FAP clearance (as observed during skeletal muscle regeneration from TNFα induced FAP apoptosis) (Lemos et al., 2015) is likely not present under these circumstance, one can speculate that this could lead FAPs to accumulate over time, contributing to increased fatty-degeneration of the skeletal muscles in obesity and T2D.

Exercise provides a unique, physiological stimulus to examine the role of mechanical and metabolic stress on skeletal muscle that results in efficient and complete repair and adaptation. Early work demonstrated that a single bout of damaging exercise increased the content of a heterogeneous population of muscle-derived mesenchymal progenitors, which likely included FAPs (Valero et al., 2012). These findings mirrored the effects of acute exercise on mesenchymal progenitors in other tissues, such as the bone marrow (Emmons et al., 2016). More recent work has suggested that the effects of exercise might be dependent on the population of mesenchymal progenitors investigated. Muscle pericytes did not increase in human muscle following eccentric exercise (De Lisio et al., 2015) or in mice following electrical stimulation (Dvoretskiy et al., 2019). This response is different from mesenchymal progenitors from other tissues, as bone marrow-derived mesenchymal progenitors are activated by an acute exercise (Emmons et al., 2016). Conversely, resistance training was associated with an increase in the content of FAPs expressing markers of active cell cycle (Farup et al., 2015). As such, the available data suggest that the effects of exercise FAP/mesenchymal progenitor activation may be dependent on the tissue of origin and specifics of the exercise stimulus, among other, yet-to-be investigated factors. Moreover, in mouse and in particular in human skeletal muscle, more in-depth phenotyping is needed in order to distinguish the specific overlapping interstitial cell populations in muscle.

## The Role of Chronic Low-Grade Inflammation in Metabolic Stress on Fap Function

The inflammatory response following muscle injury is a well-orchestrated, time-dependent process necessary to obtain complete muscle regeneration (Tidball, 2017). This response begins with the infiltration of the earliest immune cells such as neutrophils and eosinophils (Tidball, 2011). During muscle injury, IL-4 and IL-13-secreting eosinophils are recruited to the injured site (Heredia et al., 2013). These inflammatory signals act through IL-4Rα to stimulate signal transducer of transcription 6 (STAT6), which promotes FAP proliferation and inhibits FAP differentiation into adipocytes (Heredia et al., 2013). The early immune response is followed by an infiltration of macrophages with a M1 phenotype (pro-inflammatory) followed by the expansion of M2 macrophages (anti-inflammatory), which are associated with tissue repair and MuSC differentiation (Chazaud et al., 2003). Therefore, the polarization of M1 and M2 macrophages play a crucial role in successful muscle regeneration. Recently, it has been suggested that in response to acute muscle damage, macrophage-derived TNF-α plays a crucial role in regulating FAP apoptosis (Lemos et al., 2015; Fiore et al., 2016). Indeed, Lemos et al. (2015) demonstrated that in the absence of TNF-α-producing macrophages, FAPs accumulate and aberrantly differentiate into a fibrogenic lineage. Pagano et al. (2019) showed that TNF-α mediated FAP apoptosis might be perturbed in a glycerol model of muscle injury, leading to intermuscular adipose tissue development. However, treatment with the TGF-β inhibitor decorin decreases intermuscular adipose tissue development and might restore FAP apoptosis (Pagano et al., 2019). Thus, inflammatory cell-derived factors are required for proper FAP regulation, and any dysregulation of the timing or concentrations of these factors could contribute to pathological extracellular matrix accumulation by FAPs.

In contrast to the acute inflammatory response to muscle damage, myopathies, dystrophies, aging, diabetes, and obesity are associated with a chronic low-grade inflammation. This chronic, low-grade inflammation is associated impaired function of MuSCs, immune cells, and FAPs, leading to fibrosis, and poor skeletal muscle regeneration (Rostasy et al., 2008; Mann et al., 2011; D’Souza et al., 2015; Wang et al., 2015). Moreover, chronic inflammation in these conditions results in an increase in cytokine release that is responsible for the extracellular matrix production (Van Linthout et al., 2014). Consequently, muscle fibrosis develops which disrupts the cell niche for proper skeletal muscle regeneration (Murphy et al., 2011). Specifically, during chronic muscle damage, macrophage-derived TGF-β1, inhibits TNF-mediated FAP, and instead induce their fibrogenic differentiation and consequent extracellular matrix deposition (Lemos et al., 2015; Davies et al., 2016; Fiore et al., 2016; Juban et al., 2018). Similarly, Moratal et al. (2018) report that IL-1β-activated macrophages and IL-4-polarized macrophages have opposite effects on FAP differentiation into adipocytes *in vitro*, which was dependent on Smad2 phosphorylation in FAPs. Thus, under chronic inflammatory conditions associated with several metabolic disorders, signals that regulate FAP apoptosis and inhibit proliferation are perturbed, leading to a chronic state of remodeling which ultimately results in fibro/fatty tissue accumulation.

Exercise is a well-known modulator of the inflammatory response (Febbraio, 2007; Lancaster and Febbraio, 2014). In response to acute exercise, skeletal muscle produces a myriad of pro-inflammatory cytokines, including but not limited to IL-6 (Pedersen and Steensberg, 2002), IL-1ra (Ostrowski et al., 1998), IL-8, and IL-15 (Nielsen and Pedersen, 2007). The role of these cytokines in relation to skeletal muscle is to participate in the muscle repair/adaptive response to exercise (Serrano et al., 2008; McKay et al., 2009), and modulate whole-body and muscle glucose metabolism (Febbraio et al., 2004; Carey et al., 2006). Additionally, cellular mediators of the inflammatory response, increase in skeletal muscle after acute exercise (Paulsen et al., 2010), and macrophages with a pro-regenerative phenotype also increase in skeletal muscle following 12 weeks of endurance training (Walton et al., 2019). The increase in phenotypically pro-regenerative macrophages was associated with greater increases in fiber cross-sectional area and increases in MuSC content (Walton et al., 2019). Mesenchymal stromal cells in other tissues are known to regulate inflammatory responses (Munir et al., 2018); however, whether they regulate muscle inflammation, and how exercise can influence this relationship remains unknown.

## Perspectives and Conclusion

**FIGURE 1:**
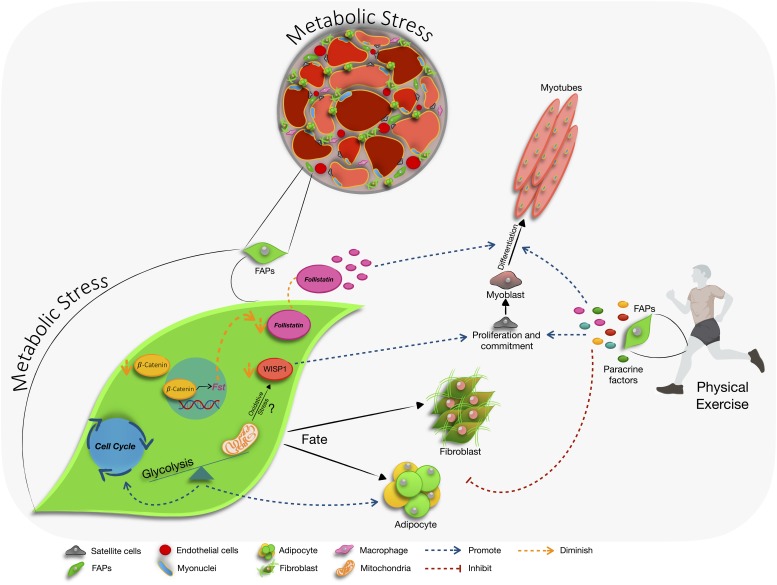
The influence of metabolic stress on fibro/adipogenic progenitor (FAPs) cell function. In skeletal muscle, metabolic stress leads to an accumulation of intermuscular adipose tissue, extracellular matrix production, and inflammation. FAPs are the primary cellular source of intermuscular adipose tissue, extracellular matrix proteins, and interact with immune cells to participate in the inflammatory response. Available literature indicates that during metabolic stress FAPs favor glycolysis during proliferation and adipogenic differentiation, and downregulate the production of the pro-myogenic factor follistatin (*fst*) via modulation of the β-catenin signaling pathway. In aging, altered secretion of WNT1 inducible signaling pathway protein 1 (WISP1) by FAPs, which may be induced by mitochondrial dysfunction, reduces their capacity to support MuSC activation and commitment. Exercise reduces the characteristic changes in skeletal muscle that occur during metabolic stress. Further, exercise stimulates FAP production of pro-myogenic factors and may inhibit adipogenic and fibrogenic potential of FAPs.

Obesity, diabetes, and other metabolic disorders are reaching epidemic proportions in western countries. Impairment of skeletal muscle is a central player in the detrimental effects of metabolic stress leading and metabolic disorders. Under conditions of metabolic stress, muscle dysfunction is characterized by excessive intermuscular adipocytes, extracellular matrix accumulation, and inflammation (Lawler et al., 2016). In these conditions, the accumulation of fibro/fatty tissue in skeletal muscle is associated with a loss of muscle mass, a reduction in muscle strength (Delmonico et al., 2009), insulin resistance, and inflammation (Szendroedi et al., 2014). As the cellular source of intermuscular adipose tissue, primary producers of the extracellular matrix, and key regulators of muscle mass, FAPs are central to these detrimental changes in skeletal muscle under metabolic stress (Figure 1). Exercise is an effective first line therapy for metabolic disorders; however, the role of exercise on FAP fate and function are just beginning to be identified. As the effects of metabolic stress and role of metabolism in regulating FAP function begin to be unraveled in the coming years, as well as the mechanisms responsible for exercise-induced FAP regulation, novel avenues for therapy will emerge to maintain muscle function, metabolic health, and reduce morbidity.

## Author Contributions

NC and MD conceived the topic for review. NC, JF, and MD wrote the review.

## Conflict of Interest

The authors declare that the research was conducted in the absence of any commercial or financial relationships that could be construed as a potential conflict of interest.
